# Oral health status and treatment needs among adults seeking dental anxiety treatment in general practice – a cross-sectional study

**DOI:** 10.2340/aos.v85.46516

**Published:** 2026-08-05

**Authors:** Mariann Saanum, Bent Schøgren Stora, Tiril Willumsen

**Affiliations:** Department of Paediatric Dentistry and Oral Health Psychology, Institute of Clinical Dentistry, Faculty of Dentistry, University of Oslo, Blindern, Oslo, Norway

**Keywords:** Dental anxiety, oral health, public health, treatment programmes

## Abstract

**Objective:**

Dental anxiety affects approximately 15% of adults worldwide and is associated with adverse oral health outcomes. The aim of this cross-sectional study was to describe oral health status and treatment needs among adults seeking dental anxiety treatment in general practice. Further, it evaluates the associations between oral health, treatment needs, age, sex, dental anxiety, and years of dental avoidance.

**Materials and Methods:**

This cross-sectional study included 88 adults with self-reported dental anxiety recruited from a rural Norwegian general dental clinic. Oral health was assessed using panoramic and intraoral radiographs combined with clinical examination. Treatment needs were evaluated according to Norwegian clinical guidelines by two dentists who reached consensus.

**Results:**

Mean age was 38.9 ± 12.2 years; 66% of the respondents were women and mean Modified Dental Anxiety Scale score was 21.0 ± 3.0. Mean decayed teeth were 5.9 ± 4.8 and missing teeth 2.5 ± 4.1. Overall, 93% of the respondents required restorative treatment, 38% endodontic treatment, and 47% extractions. Male sex and longer dental avoidance were significantly associated with poorer oral health (*p* < 0.05).

**Conclusions:**

Adults seeking dental anxiety treatment in general practice exhibit severe oral health problems comparable to those in specialist dental anxiety clinics, supporting the need for low-threshold anxiety interventions in general dental practice.

## Introduction

Dental fear and anxiety are prevalent among adults worldwide [1] and are closely associated with oral health problems in epidemiological studies [2]. In accordance with current treatment guidelines [3, 4], specialist clinics in several countries provide structured interprofessional treatment for dental anxiety [5, 6]. Evidence from these services indicates that patients with dental anxiety have poorer oral health-related quality of life (QoL), worse oral health status, and greater treatment needs compared with the general population [7–9].

Dental anxiety encompasses a spectrum of fear and apprehension related to dental treatment, ranging from mild discomfort to significant distress or dental phobia [10, 11]. Dental phobia is classified as a specific phobia under Diagnostic and Statistical Manual of Mental Disorders, 5th Edition criteria (and corresponding International Classification of Diseases, 11th Revision category 6B03), characterised by a persistent, disproportionate fear of dental treatment that may lead to marked distress and avoidance of dental care [12]. The progression from dental anxiety to dental phobia can be explained by Berggren’s vicious cycle model, which describes how anxiety drives avoidance of dental visits, leading to deteriorating oral health and increasingly complex treatment needs, thereby reinforcing and intensifying the original fear [13, 14]. Findings of a systematic review indicates that while dental anxiety affects approximately 15.0% of adults globally (95% confidence interval [CI]: 10.2–21.2), severe dental anxiety or dental phobia affects 3.3% (95% CI: 0.9–7.1) of the population [1]. This prevalence varies by demographic factors, with higher rates reported among women and younger adults [1]. Dental anxiety demonstrates consistent epidemiological patterns across different healthcare systems and cultural contexts [2, 7].

Effective management of dental anxiety is essential to address poor oral health and extensive treatment needs. Treatment approaches have traditionally been stratified by severity. Mild to moderate dental anxiety is typically managed using behavioural techniques and communication strategies within general dental practice [15, 16], whereas severe dental anxiety or dental phobia has conventionally required intervention from interdisciplinary teams comprising psychologists, dentists, and dental support staff [3]. Reports from such specialised treatment programmes indicate poor oral health at baseline, favourable treatment outcomes regarding dental anxiety reduction, and resumed dental attendance following intervention [4, 17]. However, many individuals with severe dental anxiety or dental phobia still seek care in general dental practices [18, 19], potentially owing to limited access to specialist services or personal preferences regarding treatment setting.

A knowledge gap exists regarding the characteristics of patients with severe dental anxiety who receive treatment in general dental clinics. It remains unclear whether the extensive treatment needs reported in specialist dental anxiety clinics reflect the needs of patients managed in general practice. Hauge et al. previously reported reductions in dental anxiety in a randomized controlled trial (RCT) evaluating two treatments for dental anxiety in general practice [20], but the oral health characteristics of the trial participants have not yet been described. Earlier intervention to prevent oral health deterioration may be more feasible in general practice than in specialist services, where geographical distance, waiting lists, and intake restrictions can limit access to care. In this context, it is important to describe the oral health status and in particular, the dental treatment needs of adults with established dental anxiety who seek care in general practice, such as those included at baseline in Hauge et al.’s RCT. Doing so will help to fill this knowledge gap and provide clinically relevant information for general dental practitioners managing patients with dental anxiety.

It was hypothesised that patients seeking dental anxiety treatment in general practice would exhibit fewer oral health problems and lower treatment needs than those reported in specialist clinic populations.

This study aimed to describe oral health status and dental treatment needs among adults seeking dental anxiety treatment in general practice through: (1) describing relevant oral health variables; (2) describing dental treatment needs as assessed by clinical dentists; (3) evaluating associations between oral health, treatment needs, age, sex, dental anxiety, and years of dental avoidance; and (4) situating the observed oral health and dental treatment needs in relation to findings from recent Norwegian population studies and from specialist clinics for dental anxiety, in order to provide contextual interpretation.

## Materials and methods

This cross-sectional descriptive study reports baseline oral health data from participants enrolled in an RCT comparing two treatments for dental anxiety in general practice: dentist-administered cognitive behavioural therapy (D-CBT) [21], and the Four Habits Model [22], combined with oral conscious sedation with midazolam (Four Habits+midazolam). A full description of the interventions is provided in the primary RCT publication [20].

The study was conducted at a general dental practice in Mandal (Agder County), a mid-sized city in southern Norway with adequate dentists in public and private sectors. National register data on adult oral health are unavailable; however, among 18-year-olds, Agder ranks fourth among Norwegian counties for caries experience (32.7% caries-free vs. 26.7% national average [23]). The trial was approved by the Norwegian Regional Committee for Medical and Health Research Ethics (ID: 2017/97) and registered at ClinicalTrials.gov (identifier: NCT03293342).

### Inclusion criteria

Participants were eligible for inclusion if they self-reported dental anxiety of sufficient severity to interfere with their ability to undergo dental treatment. There was no minimum threshold for dental anxiety; eligibility was based on self-reported dental anxiety without reference to any specific score on dental anxiety scales. The Modified Dental Anxiety Scale (MDAS) and The Index of Dental Anxiety and Fear (IDAF-4C) were administered at baseline as part of the study assessments, not as screening tools. Additional inclusion criteria included being 18 years of age or older and having the ability to communicate fluently in Norwegian.

### Exclusion criteria

Participants were excluded if they had current substance abuse (due to potential interactions with the treatment drug) or severe mental/neuropsychiatric conditions precluding participation in D-CBT.

### Patient sample and recruitment method

Participants were invited through advertisements in social media and a local newspaper, as well as through referrals from nearby dentists. A research assistant provided written and oral information about the study and obtained written informed consent prior to enrolment. In total, 96 individuals agreed to participate between September 2017 and March 2020. The majority of the self-reported data were collected using a paper questionnaire completed by participants in the dental clinic prior to randomisation and enrolment in the RCT. The research assistant who distributed the questionnaire remained available to answer questions during completion. In all, 88 participants attended the first clinical appointment with oral health registrations, which coincided with the first session of the dental anxiety intervention, and were consequently included in the present baseline analyses. During the initial clinical interview, information about dental avoidance (operationalised as ‘self-reported number of years since the dentition was last fully restored’) was obtained for 78 participants, while 10 did not provide this information. Consequently, analyses of this variable included 78 participants. Approximately three quarters of the participants were self‑referred, while the remainder were referred by local dentists. Most participants lived in Mandal or the surrounding area within a 15 km radius. All dental anxiety treatments and related dental care provided within the study were free of charge for the participants. Specific reasons for non‑attendance were not systematically recorded.

### Data collection

Data were collected using panoramic radiographs (orthopantomograms), supplemented by intraoral radiographs (bitewing and periapical images), as well as a standardised series of six clinical photographs. Two registration forms, adapted from those used in a previous study conducted at a specialist dental anxiety clinic [8], were developed to assess oral health status and dental treatment needs (Appendices 1 and 2). The first author (MS) conducted a clinical evaluation of each participant. All assessments followed written diagnostic criteria adapted from the registration forms developed for the previous specialist dental anxiety clinic study. In collaboration with a second dentist, the assessments followed a two-step consensus procedure. First, the two dentists independently assessed diagnoses and treatment needs according to the standardised registration forms (Appendices 1 and 2). Second, they met to compare their assessments item by item. In cases of disagreement, the relevant panoramic and intraoral radiographs, standardised clinical photographs, and electronic patient journal records were jointly reviewed and discussed until full consensus was reached. No formal inter-rater reliability statistic (e.g., Cohen’s kappa or intraclass correlation coefficient) was calculated.

### Measures

Background variables included age (continuous, years), sex (binary), and avoidance of dental care -operationalised as self-reported years since the dentition was last fully restored (continuous, years).

Dental anxiety was assessed using the MDAS, developed by Humphris et al. [24] as an extension of Corah’s four-item Dental Anxiety Scale [25]. The MDAS includes an additional fifth item addressing injection-related anxiety [26]. A cutoff score of ≥19 on the MDAS has been widely used to operationalise severe dental anxiety or dental phobia and demonstrates high sensitivity (87.5%) and specificity (89.3%) for identifying individuals with phobic avoidance patterns [24]. This validated threshold has been applied to distinguish severe dental anxiety from moderate anxiety, with individuals scoring ≥19 typically exhibiting marked avoidance of dental care and significant functional impairment [26]. The brief format of the MDAS – five questions requiring approximately 2–3 min to complete – makes it well suited for clinical settings, and its established cutoff scores and widespread use facilitate comparisons across international studies [26].

The IDAF-4C was included as a secondary measure to complement the MDAS, as it captures cognitive, emotional, behavioural, and physiological components of dental anxiety separately, and to facilitate comparison with recent international studies employing this instrument. Developed by Armfield [10], the IDAF-4C adopts a theory-based framework that separately assesses cognitive, emotional, behavioural, and physiological components of dental anxiety and fear through eight items, with two items per component. The scale demonstrates excellent psychometric properties, including a high internal consistency and strong test–retest reliability across diverse populations [10, 27]. Responses are recorded on a 5-point Likert scale and standardised by dividing the total score by the number of items, with scores of ≥3.0 indicating dental anxiety. The IDAF‑4C used in this study was translated into Norwegian by bilingual dental clinicians and back‑translated into English for checking but has not yet been subjected to a full psychometric validation in a Norwegian population. Validation relies on studies of the original and translated versions, which report good reliability and validity in adult samples from other Nordic and European countries [27, 28]. The IDAF-4C effectively predicts dental attendance patterns and avoidance behaviours [10].

Oral health-related QoL was assessed using the Oral Impacts on Daily Performance (OIDP) index. Developed by Adulyanon et al. [29], the OIDP evaluates the impact of oral health on eight daily activities over the previous 6 months: eating, speaking, cleaning teeth, sleeping, smiling, maintaining emotional well-being, social interaction, and performing work or social roles. Eight questions are answered on a 5-point scale to assess the frequency of such impacts on daily tasks in each area; the answers are reverse-scored, ranging from 5 (‘every day’) to 1 (‘never’). Total scores range from 8 to 40, with higher scores indicating that oral issues have a more negative impact on daily life. The OIDP has been validated in Norwegian populations [30] and demonstrates satisfactory psychometric properties, including a moderate test–retest reliability (Cohen’s κ = 0.65) and high internal consistency (Cronbach’s α ≥ 0.80).

#### Oral health registrations

The number of decayed teeth was recorded as teeth with primary or secondary caries extending into the dentine (D3–D5). Missing teeth were defined as the total number of absent teeth, and filled teeth were defined as teeth containing one or more restorations. Marginal bone loss was assessed radiographically and recorded at two severity levels: (1) advanced marginal bone loss, defined as a marginal bone level more than 4 mm apical to the cement–enamel junction but coronal to the apical third of the root, and (2) total marginal bone loss, defined as a marginal bone level extending into the apical third of the root. The number of endodontically treated teeth was recorded, including teeth present only as root remnants. Root remnants were defined as severely destroyed teeth that could not be restored; multi-rooted teeth were recorded as a single tooth. Apical radiolucencies were recorded as the number of teeth, excluding root remnants, showing pathological alteration of the lamina dura and radiographic evidence of apical radiolucency.

#### Dental treatment needs

Dental treatment needs were assessed in accordance with the Norwegian standard ‘Good clinical practice in the dental services’ [31]. Third molars were included only when extractions were indicated; otherwise, they were excluded from treatment need counts. Restorative treatment needs were recorded as the number of teeth requiring fillings or crowns. Teeth in need of endodontic treatment were defined as restorable teeth presenting with periradicular bone lesions and/or clear pulp involvement. The need for tooth replacements in the anterior or premolar region was recorded as the number of missing teeth or teeth requiring extraction mesial to the first molars, including incisors, canines, and premolars. Denture needs were recorded as the number of new dentures required. Extractions were defined as root remnants, teeth with marginal bone loss extending into the apical third of the root, third molars with signs of pathological findings, and partially erupted or impacted teeth with evident communication with the oral cavity. Periodontal status was assessed clinically and radiographically as part of routine examinations, but no systematic full‑mouth periodontal charting (probing pocket depths, clinical attachment levels or bleeding on probing) was performed. As these parameters are essential for determining current disease activity and periodontal treatment need, the need for periodontal treatment could not be reliably estimated from the available data.

### Sample size

The sample size calculation for the original RCT study was based on detecting a between‑group difference of 20% in change in MDAS scores with 80% power at a two‑sided significance level of 0.05 [20]. No additional, study-specific sample size calculation was performed for this cross-sectional baseline analysis.

### Statistical analysis and variable coding

Stata SE 18.0 (StataCorp LLC, College Station, TX, USA) was used for all analyses. Descriptive statistics were used to summarise sample characteristics, oral disease, treatment needs, and scores on MDAS, IDAF‑4C and OIDP. IDAF‑4C and OIDP were analysed descriptively to characterise the psychological and functional burden associated with dental anxiety and oral disease in the sample and were not included as independent variables in regression models.

Logistic regression analyses were performed with three dichotomous dependent variables: any apical radiolucency, any root remnant, and any need for tooth replacement in the anterior/premolar region. These variables were dichotomised as any versus none, due to small cell counts. Independent variables in all models were sex, age, dental anxiety (measured by MDAS), and dental avoidance. Age and dental avoidance were each dichotomised at the sample median (age ≥ 37 years; avoidance ≥ 7), to ensure adequate group sizes given the relatively small sample (*n* = 88). Adjusted odds ratios (OR) with 95% confidence intervals (CI) were estimated with mutual adjustment for these four independent variables. For MDAS, the established clinical cut-off of ≥ 19 was not used, as 84% of participants scored at or above this threshold, resulting in insufficient variation for meaningful regression analyses; instead, MDAS was dichotomised at the sample median (≥ 22) to achieve more balanced group sizes. Covariates were selected based on prior literature and clinical relevance, not stepwise procedures, to identify easily observable risk indicators rather than build a prediction model.

## Results

Of 96 participants admitted to the study, 8 dropped out before completion of the clinical registrations and 10 did not provide data on dental avoidance. Thus, 88 datasets were available for descriptive analyses. No statistically significant differences were observed between those who completed oral registrations and those who did not in terms of sex, age, dental anxiety, or years since the dentition was last fully restored (Appendices 3 and 4 for study flow and dropout details). Despite the absence of threshold inclusion criteria for anxiety, 74 participants (84%) reported high dental anxiety with a MDAS score of 19 or more. Participant characteristics are presented in Table 1.

**Table 1:** Sample characteristics (n = 88): demographics, dental anxiety, oral health related quality of life, and avoidance behaviour.

Background variables	*n* = 88
Sex (female percentage)	58 (65.9%)
Age (mean (SD)/(range))	38.9 (12.2)
	(19–65)
Years since last complete dental treatment (mean years (SD)/(range))	9.9 (9.8)
	(0–40)
MDAS (mean sum score (SD)/(range))	21.0 (3.0)
	(12–25)
IDAF 4C (mean score (SD)/(range))	4.2 (0.6)
	(2.6–5.0)
OIDP (mean sum score (SD)/(range))	19.6 (8.8)
	(8–40)

MDAS: Modified Dental Anxiety Scale; IDAF-4C: Index of Dental Anxiety and Fear; OIDP: Oral Impact on Daily Performances; SD: standard deviation.

Values are presented as *n* (%) for the binary variable (sex) and as mean (SD) with range in parentheses on the line below for remaining continuous variables.

### Oral health

The mean number of teeth present with a clinical crown was 24.8 (standard deviation [SD]: 5.0; range: 4–28). Eleven participants (12.5%) had 20 or fewer teeth remaining, whereas 37 (42.0%) retained all 28 teeth. The sample was characterised by high prevalence of caries, root remnants, apical radiolucencies, and missing teeth (Table 2). The mean number of decayed teeth (D3–5T) was higher in men than in women and was highest in the 25–54-year age groups, with lower mean values in the youngest (19–24 years) and oldest (55+ years) participants (Table 3).

**Table 2:** Oral health status (n = 88): decayed, missing, filled teeth, bone loss, endodontic status, and peri-radicular bone lesions.

Oral health variable	Participants with ≥1 affected tooth, n (%)	Number of affected teeth per participant, Mean (SD)		
	*N* = 88 (100%)	Mean (SD)	Median	Range
Decayed	79 (89.8%)	5.9 (4.8)	4	0–24
Filled	33 (37.5%)	7.8 (5.2)	8	0–24
Missing	46 (52.3%)	2.4 (3.9)	1	0–24
Endodontically treated	26 (29.5%)	0.6 (1.5)	0	0–7
Peri-radicular bone lesions	31 (35.2%)	0.5 (1.0)	0	0–5
Advanced bone loss	22 (25.0%)	1.9 (4.6)	0	0–20
Root remnants	26 (29.5%)	1.4 (3.1)	0	0–16

SD: standard deviation.

The number of root remnants and missing teeth, as well as decayed teeth (teeth with caries extending into the dentine), fillings, endodontically treated teeth, peri-radicular bone lesions (pathological alteration of the lamina dura and radiographic evidence of apical radiolucency), and advanced bone loss (defined as a marginal bone level more than 4 mm apical to the cement–enamel junction), are specified along with the corresponding range and median values. The number and of patients affected (out of a total of 88 patients) is listed in the first column (percentage between parenthesis).

**Table 3:** Oral health status (n = 88): decayed, missing and filled teeth, stratified by age and sex.

Participant group	*N*	D3-D5 T Mean (SD) (Range)	MT Mean (SD) (Range)	FT Mean (SD) (Range)
All	88	5.9 (4.8) (0–24)	2.4 (3.9) (0–24)	7.8 (5.2) (0–24)
Men	30	8.0 (5.6) (0–24)	2.2 (3.0) (0–10)	6.1 (4.5) (0–17)
Women	58	4.8 (3.9) (0–15)	2.4 (4.3) (0–24)	8.7 (5.3) (0–24)
Age (years)				
19–24	7	4.4 (3.8) (0–10)	0.9 (1.1) (0–2)	7.4 (5.3) (1–17)
25–34	32	8.0 (5.3) (0–24)	0.6 (1.1) (0–5)	7.4 (4.6) (0–18)
35–44	18	5.7 (4.7) (0–18)	1.6 (2.2) (0–7)	5.9 (4.2) (0–14)
45–54	19	5.1 (4.0) (0–14)	4.9 (6.1) (0–24)	8.6 (4.1) (0–17)
55–64	10	2.8 (2.9) (0–9)	5.9 (4.0) (0–11)	8.6 (7.3) (0–24)
65–74	2	0.5 (0.7) (0–1)	1.5 (0.7) (1–2)	20.5 (0.7) (20–21)
75+	0	-	-	-.

SD: standard deviation.

Table showing the mean number of teeth with caries lesions extending into dentine (D3–D5 T), missing (MT), and filled (FT) teeth, stratified by age and sex. Standard deviations and ranges are given in parentheses.

### Dental treatment needs

Participants exhibited extensive dental treatment needs, with restorative treatment required by more than 90% of patients (*n* = 82/88) (Table 4). Ten participants (11.4%) required extraction of six or more teeth, and dental caries was the main reason in all cases. At baseline, three participants (3.4%) required one removable denture and five (5.7%) required two removable dentures. Kennedy classification was used to describe the partially edentulous situations, where Class I denotes bilateral free-end saddles, Class II unilateral free-end saddles, and Class III bounded edentulous spaces. One of the single-denture cases corresponded to a Kennedy Class II situation and two to Class I, whereas among those needing two dentures, four required one Kennedy Class I and one Class II denture, and one participant required two Class I dentures.

**Table 4:** Dental treatment needs (n = 88): restorations, endodontics, extractions, replacements, and dentures.

Dental treatment need	Participants with ≥1 affected tooth, n (%)	Number of affected teeth per participant, Mean (SD)		
	*N* = 88 (100%)	Mean (SD)	Median	Range
Restorative treatment	82 (93.2%)	5.9 (4.6)	4.5	0–20
Endodontic treatment	33 (37.5%)	0.6 (0.9)	0	0–4
Extractions	41 (46.6%)	1.8 (3.3)	0	0–17
Replacements	33 (37.5%)	1.3 (2.6)	0	0–13
Dentures	8 (9.1%)	-	0	0–2

SD: standard deviation.

In Table 4, Dental treatment needs are specified at the patient level (Participants) with percentages in parentheses and at the tooth level (Teeth) with standard deviations in parentheses, followed by the median and range at the tooth level. ‘Replacements’ is referred to need for tooth replacement in the front and premolar region.

### Oral health and dental treatment needs in relation to sex, age, dental anxiety, and years of avoidance

Ten participants were unable to recall when they last completed dental treatment; therefore, 78 datasets were available for regression analyses involving this variable. In both univariate analyses and fully adjusted logistic regression models, male sex and longer avoidance duration were significantly associated with higher odds of having teeth requiring replacement, periradicular bone lesions, or root remnants (Table 5). Age was also an important predictor, although its association did not reach statistical significance for root remnants in univariate analyses or for periradicular bone lesions in multivariate analyses.

**Table 5:** Risk factors related to oral health and treatment needs (n = 78): unadjusted and adjusted odds ratios for gender, age, the Modified Dental Anxiety Scale score, and years since last treatment.

Dependent variable	Independent variables	Unadjusted effects			*P*	Adjusted effects			*P*
		OR	Z	95% CI		OR	Z	95% CI	
Replacement	Gender (male)	3.43	2.62	1.36–8.54	0.009	6.11	2.71	1.65–22.61	0.007
	Age > 39	3.16	2.52	1.29–7.76	0.012	3.72	2.22	1.15–11.99	0.028
	MDAS ≥ 22	2.62	2.12	1.08–6.40	0.034	3.16	1.83	0.92–10.86	0.067
	7 years or more since last complete dental treatment	5.46	3.26	1.97–15.15	0.001	4.16	2.46	1.33–12.97	0.014
Root remnants	Gender (male)	3.39	2.72	1.41–8.18	0.007	7.23	4.88	1.92–27.14	0.003
	Age > 39	1.91	1.52	0.83–4.41	0.129	3.67	2.11	1.10–12.25	0.034
	MDAS ≥ 22	1.99	1.62	0.87–4.60	0.105	3.34	1.85	0.93–11.97	0.064
	7 years or more since last complete dental treatment	6.30	3.4	2.18–18.17	0.001	5.4	2.74	1.61–18.08	0.006
Radiolucencies	Gender (male)	3.27	2.51	1.29–8.28	0.012	3.31	2.06	1.06–10.32	0.040
	Age > 39	2.56	2.05	1.04–6.28	0.040	2.98	1.95	0.99–8.96	0.052
	MDAS ≥ 22	0.97	−0.07	0.40–2.33	0.947	0.84	−0.30	0.28–2.57	0.763
	7 years or more since the dentition was fully restored	4.49	2.89	1.62–12.45	0.004	3.53	2.25	1.18–10.54	0.024

MDAS: Modified Dental Anxiety Scale; OR: odds ratio; Z: Wald statistic for testing the null hypothesis that the coefficient equals zero; 95% CI: 95% confidence interval; *P*-value: Statistical significance.

Logistic regression analyses of associations between independent variables (sex, age, MDAS, years since last complete dental treatment) and oral health/treatment need outcomes including the dependent variables: Replacement = ‘Need for teeth replacement in the anterior/premolar region of the mouth’, Root remnants = ‘Presence of root remnants’ and Radiolucencies = ‘Apical radiolucencies’, as well as the independent variables ‘Gender’ (male), ‘Age’ (being 40 years or more), MDAS-score (score of 22 or above) and ‘7 years or more since the dentition was fully restored’ meaning that all oral disease had been treated and good functional status achieved. Statistically significant findings are in bold.

Higher dental anxiety, measured by MDAS-score, predicted tooth replacement needs in the anterior/premolar region in univariate analyses only. After adjustment for age, sex, and years of avoidance, the MDAS score was no longer significantly associated with replacement needs. Dental anxiety severity was not significantly associated with root remnants or periradicular bone lesions in either univariate or multivariate models (Table 5).

The multivariate logistic regression models explained 16.4% of the variance in the presence of apical radiolucencies, 24.5% of the variance in the presence of root remnants, and 27.5% of the variance in the need for tooth replacement in the anterior or premolar region.

### Comparison with population-based studies and specialist clinics

Oral health status and dental treatment needs in the present sample were comparable to findings reported in specialist dental anxiety clinics but differed from those observed in general population-based studies (Table 6).

**Table 6:** Caries prevalence compared to Norwegian population studies and specialist dental anxiety clinics.

Study / population	Mean	SD	Min	Max	*N*
This study	5.9	4.8	0	24	88
Agdal et al. (2008)	6.7	4.3	1	21	40
Westad et al. (2024)	6.6	5.5	1	24	20
Rødseth et al. (2023)	1.4	≈1.7			4,880
Oscarson et al. (2017)	1.1	≈1.5			1,932

SD: standard deviation.

This table compares the mean caries prevalence (D in DMFT) in the present study with those from specialist clinics and the general population. The mean is reported, as well as the SD, minimum and maximum values (min/max) when available, and the number of participants (*N*).

## Discussion

Participants in the present study reported severe dental anxiety and presented with extensive oral disease, extensive unmet treatment needs, and reduced oral health-related QoL. Most required restorative treatment, endodontic treatment, and extractions (93%, 38%, and 47%, respectively), and more than one 3^rd^ required tooth replacement in the anterior or premolar region. Male sex and a longer history of dental avoidance was associated with poorer oral health outcomes. Contrary to our hypothesis, oral health status and treatment needs among adults seeking dental anxiety treatment in general dental practice were comparable to those previously reported in specialist dental anxiety clinics. The findings suggest that severe dental anxiety and long‑term avoidance can lead to a level of oral disease burden that is similar irrespective of whether patients ultimately seek help in general or specialist settings.

The participants exhibited significantly poorer oral health compared with individuals in recent Norwegian general population-based studies. For example, the prevalence of caries was approximately 3–4 times higher, compared with mean decayed teeth of 1.4 (95% CI: 1.3–1.4) reported by Rodseth et al. [32] and 1.1 (95% CI: 1.0–1.2) reported by Oscarson et al. [33]. This difference likely reflects a combination of selection bias inherent to a treatment-seeking sample and the well-documented impact of dental avoidance on cumulative oral disease burden. Although direct comparisons between a treatment-seeking clinical sample and general population data must be interpreted cautiously, the observed differences in oral health status are nonetheless informative. Oral health status was however comparable to that reported in patients seeking treatment in specialist dental anxiety clinics, with the mean number of decayed teeth in the present study (5.9 ± 4.8) closely matching findings by Westad (6.6 ± 5.5) [9] and Agdal (6.6 ± 4.2) [8]. The high comparability of the present study with the study conducted by Agdal et al. is strengthened by the use of identical clinical registration schemas. This pattern of pronounced oral health deterioration aligns with international evidence from populations with dental anxiety. Heidari et al. reported that among 10,900 participants in the 2009 Adult Dental Health Survey, 12% had dental phobia, with higher odds of having one or more decayed teeth and increased likelihood of missing teeth [2]. This finding may reflect the progression from untreated caries to tooth loss over time due to avoidance behaviour – a pattern consistent with both the present findings and those of longitudinal research conducted by Schuller et al. [34], highlighting the critical importance of early intervention to prevent irreversible oral health decline.

Being female and demonstrating fewer years of avoidance were the only independent variables significantly negatively associated with the dependent outcomes (root remnants, periradicular bone lesions, and the need for tooth replacement in the anterior or premolar region) after adjustment for age and dental anxiety severity. Previous research has also documented sex-related differences in oral health outcomes [35], supporting the associations observed in the present study. Given that oral disease progresses when left untreated, the association between longer avoidance periods and greater oral health deterioration and more severe treatment needs was expected. In the present study, self-reported dental anxiety served as the inclusion criterion. Accordingly, the mean MDAS score was high (21.0 ± 3.0), with 84% of participants scoring at or above the phobia threshold of 19. This restricted variability in anxiety severity may explain why the MDAS score did not predict oral health outcomes in this sample, in contrast to findings from population-based studies, where a broader distribution of anxiety levels is observed [36].

Patients with self-reported dental anxiety were recruited via social media, local advertisements, and dentist referrals, with no anxiety severity threshold applied. Agder County ranks among the Norwegian counties with more favourable outcomes on adolescent caries experience (32.7% caries-free vs. 26.7% nationally), and this pragmatic recruitment approach in general practice suggests that the baseline data are representative of treatment needs among anxious adults in typical Norwegian general practice. This enhances the external validity of the study and represents an important methodological strength.

Important limitations should nevertheless be acknowledged. First, recall bias may have affected self-reported measures, particularly the estimate of years of avoidance. Second, participant tolerability affected the availability and quality of intraoral radiographs and photographs. This variation may have influenced diagnostic accuracy, given that panoramic radiographs alone are insufficient for reliable detection of certain oral pathologies. Panoramic radiographs reliably detect missing teeth and retained root remnants [37] but are limited for caries and detailed periodontal assessments [38]. The use of complementary clinical registrations, photographic documentation, and structured consensus discussions likely strengthened diagnostic reliability. Third, the dentists responsible for data collection were not calibrated, either among themselves or against an external standard, and no inter-rater reliability statistics (such as Cohen’s kappa or the intraclass correlation coefficient) were calculated. Agreement instead relied on the two-step consensus procedure described in the Methods section, in which discrepancies were resolved through joint re-evaluation of radiographs, photographs, and clinical records. This lack of formal calibration may have affected diagnostic consistency, particularly for more subjective assessments such as caries grading and periapical pathology. Fourth, the study sample comprised adults with self‑reported dental anxiety who actively sought treatment and were either self‑referred or referred by local dentists to a single general dental practice. This treatment‑seeking clinical sample does not represent the general population. Consequently, comparisons with population‑based studies are descriptive only; the population data are used to contextualise the clinical relevance of the observed burden of oral disease and treatment needs in this group, rather than support direct generalisations. Finally, the IDAF-4C has not yet been formally validated in a Norwegian adult population; although Nordic and European studies indicate good psychometric properties, this limitation should be considered when interpreting findings based on this instrument.

Previous findings in the present study sample [39] demonstrated significant reductions in symptoms of generalised anxiety—as well as reductions in post-traumatic stress symptoms following successful treatments for dental anxiety. Improvements in oral health-related QoL were also observed, indicating meaningful spillover effects on overall well-being. These findings align with a growing body of evidence highlighting the substantial impact of oral health on general health and QoL [40].

The present study contributes to the existing evidence base on dental anxiety and oral health outcomes in general dental practice. Although participants in this cross-sectional study were recruited from a population characterised by generally good oral health, they nevertheless presented with considerable oral disease and unmet treatment needs at baseline. Following targeted interventions for dental anxiety, most participants were able to engage in regular dental care [41]. These findings underscore that reducing dental anxiety, when present, should constitute an initial and essential step in the long-term management of extensive oral disease.

In conclusion, the comparable oral health deterioration observed across general dental practice and specialist anxiety clinics reveals that untreated dental anxiety exerts a measurable and clinically significant toll irrespective of care setting – one that extends beyond oral health to encompass emotional, financial, and social consequences for affected individuals. This burden is neither confined to specialised populations nor dependent on care context – it is systemic. Prioritising the implementation of low-threshold anxiety interventions within general dental practice thus represents both a clinical necessity and a public health opportunity.

Future research should assess the effectiveness, feasibility, and cost implications of existing treatment programmes. Further investigation is also needed to determine the long-term impact of these interventions on oral, general, and mental health, as well as on overall QoL.

## Supplementary Material



## Data Availability

The data supporting the findings of this study are available from the corresponding author (MS) upon reasonable request.
