# Supplementary material for: Oral health status and treatment needs among adults seeking dental anxiety treatment in general practice – a cross-sectional study
Source: Acta Odontol Scand. 2026 Aug 5;85:46516. doi: 10.2340/aos.v85.46516 (PMC13454916; doi:10.2340/aos.v85.46516)

Supplementary material has been published as submitted. It has not been copyedited or typeset by Acta Odontologica Scandinavica.

Appendix 1

Project number		Date	

	Number
Teeth in need of a filling or crown	
Teeth in need for periodontal treatment	
Teeth in need of endodontic treatment	
Teeth that need replacement (1-5)	
Number of dentures needed	
Teeth that need extraction	
Total number of teeth in need of treatment	

Appendix 2

Project number [ ] Date [ ]

	Number
Teeth in need of a filling or crown	
Teeth in need for periodontal treatment	
Teeth in need of endodontic treatment	
Teeth that need replacement (1-5)	
Number of dentures needed	
Teeth that need extraction	
Total number of teeth in need of treatment	

Appendix 3 Characteristics of completers versus drop-outs	**Completers**	**Drop-outs**	
	**n=88**	**n=8**	**Chi-sqare**
**Sex (female percentage)**	58 (65.9%)	5 (62.5%)	p=0.846
			**T-test**
**Age (SD)**	38.9 (12.2)<br>(19-65)	33.5 (12.4)<br>(22-57)	p=0.115
**Years since last full dental treatment**	9.9 (9.8)<br>(0-40)	8.7 (7.1)<br>(1-22)	p=0.765
**MDAS**	21.0 (3.0)<br>(12-25)	21.1 (1.5)<br>(19-23)	p=0.908

Appendix 3 shows the proportion of women compared to men that participated in the study, the mean age of participants and dental anxiety measured by both the modified dental anxiety scale (MDAS). The values are compared between completers and drop-outs by using chi-square test on binominal data and t-test on ordinal data.

Appendix 4 Study-flow

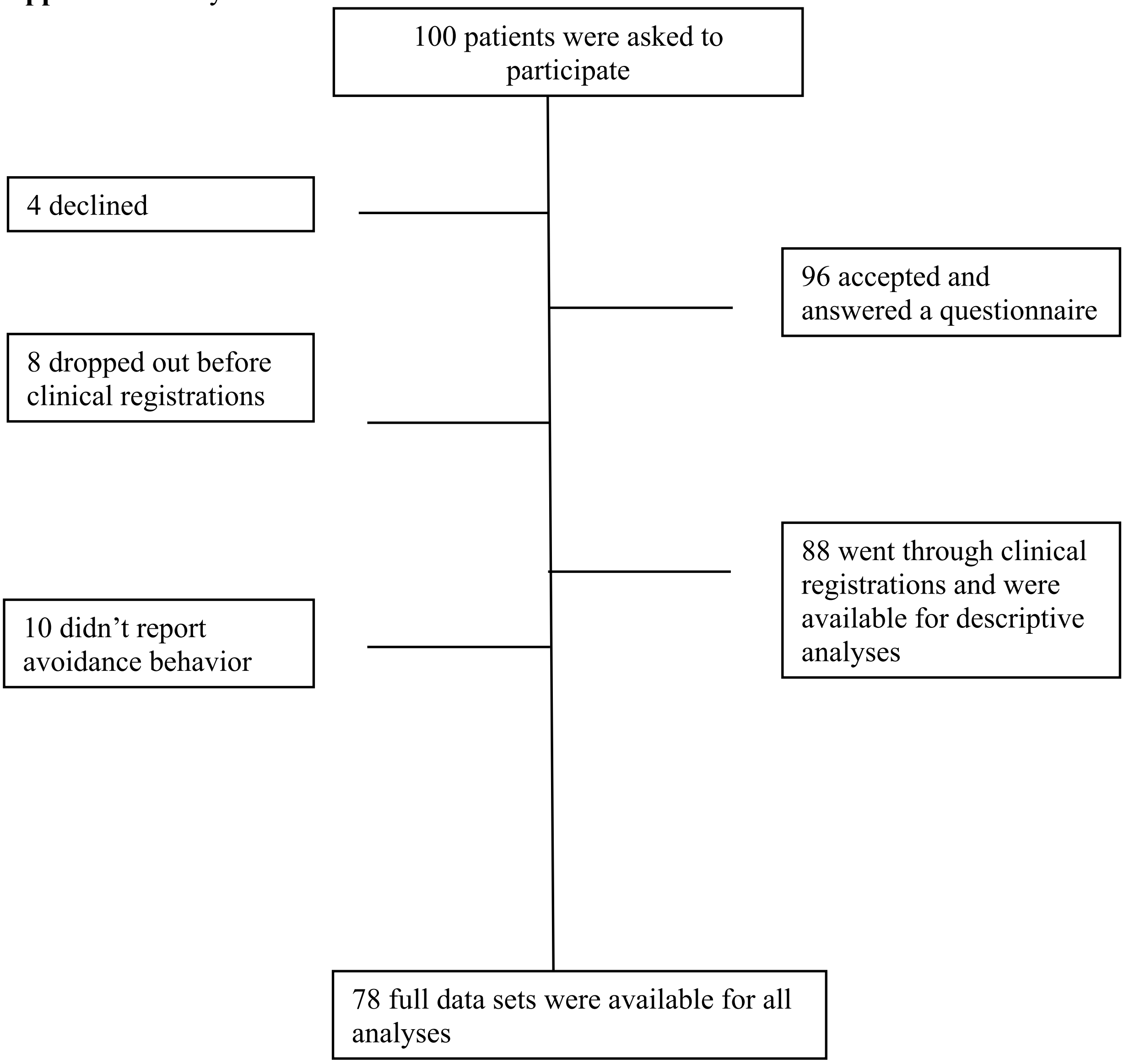

Supplement: Supplementary file 1 [file AOS-85-46516-s1.pdf]
